# Areolar leiomyoma: clinical and histopathological aspects of two cases^؆^

**DOI:** 10.1016/j.abd.2025.501217

**Published:** 2025-10-27

**Authors:** Mariana Abdo de Almeida, Neusa Yuriko Sakai Valente, Thaiz Brandão Cosac

**Affiliations:** a Department of Pathological Anatomy, Instituto de Assistência Médica ao Servidor Público Estadual, São Paulo, SP, Brazil; b Department of Dermatology, Hospital do Servidor Público Estadual de São Paulo, São Paulo, SP, Brazil

Dear Editor,

Cutaneous leiomyomas are rare benign smooth muscle tumors. These tumors are most commonly reported in middle-aged women, with an approximate female-to-male sex ratio of 3:1.6. These lesions are differentiated based on the cell of origin and classified as pilar leiomyoma, angioleiomyoma, and genital leiomyoma. The objective of this study is to report two cases of areolar leiomyomas and discuss their diagnostic aspects.

## Case 1

**Figure 1:**
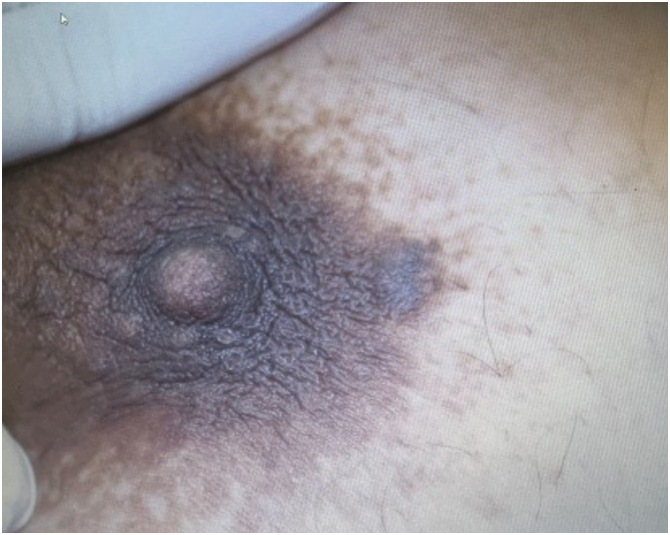
Patient in Case 1. Normochromic papule in relation to the mammary areola tissue, measuring approximately 5 mm, with regular contours and well-defined, located on the lateral margin of the areola.

A 50-year-old woman was sent by a dermatologist to a referral center with a request for a biopsy of a lesion in the left areola. The patient had no known comorbidities and denied associated symptoms such as pain, bleeding, or discharge. Physical examination revealed a rounded, nodular lesion measuring approximately 0.5 cm in diameter, with an irregular surface and brown color, located on the lateral-superior border of the left areola (Fig. 1). Dermoscopy revealed a whitish-yellowish central area and a discreet pigmented network in the periphery. The diagnostic hypothesis of an epidermal cyst or dermatofibroma was raised.

## Case 2

A 68-year-old woman presented with a lesion in the left areola without associated symptoms. Physical examination revealed a nodular, hyperchromic lesion located at 10 o'clock in the left areola. The diagnostic hypothesis of epidermal cyst or scar fibrosis was raised.

**Figure 2:**
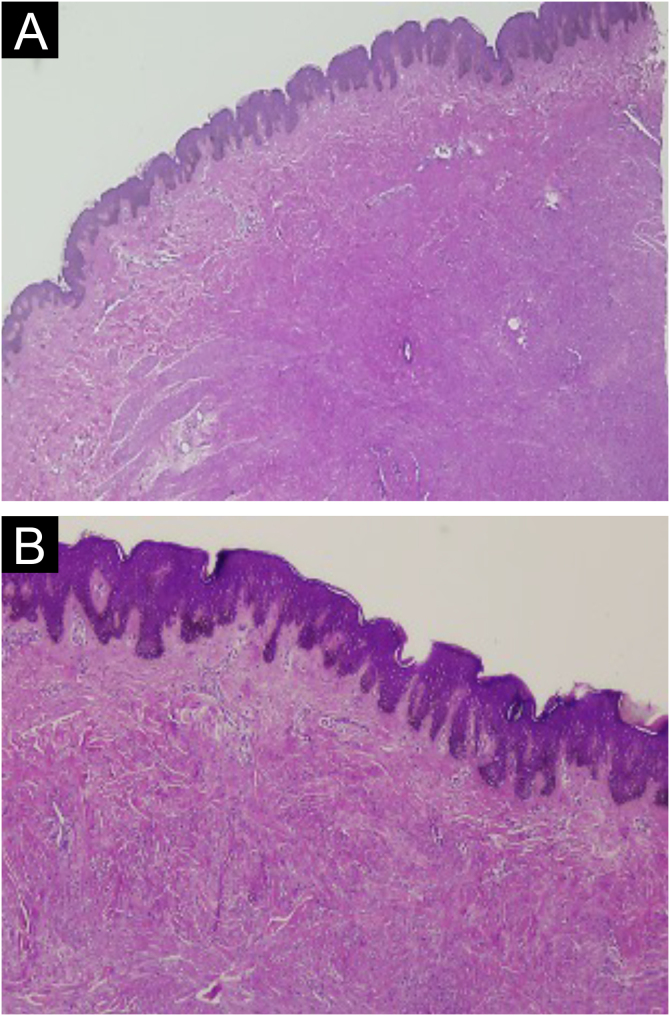
Case 1. (A) Circumscribed dermal proliferation of monotonous smooth muscle cells (Hematoxylin & eosin, ×20). (B) Intertwined bundles of smooth muscle cells with scant intervening connective tissue (Hematoxylin & eosin, ×40).

**Figure 3:**
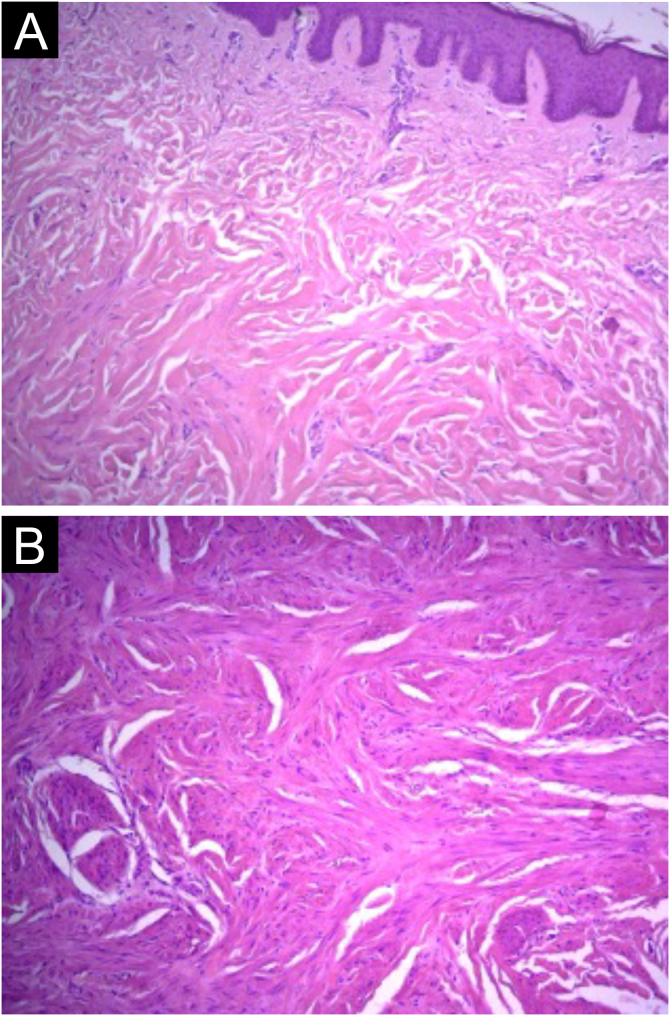
Case 2. (A) Proliferation of smooth muscle cells showing intersecting bundles (Hematoxylin & eosin, ×100). (B) Cells with elongated nuclei, eosinophilic cytoplasm, without significant atypia or mitotic figures (Hematoxylin & eosin, ×100).

**Figure 4:**
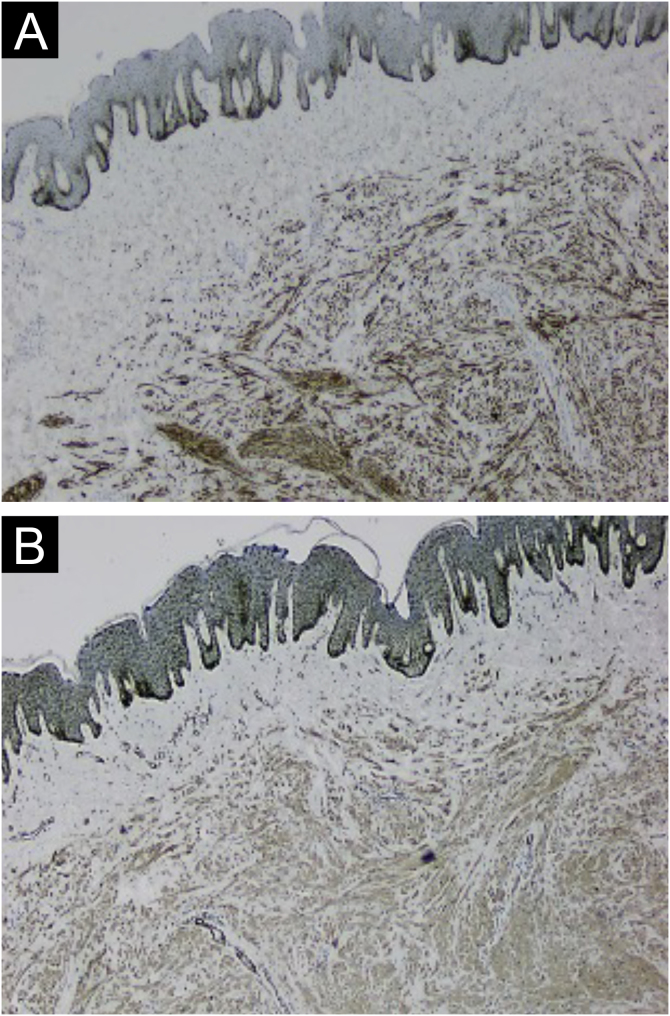
Case 1. (A) Immunohistochemistry with desmin antibody showing positivity in neoplastic cells (×40). (B) Immunohistochemistry with smooth muscle actin antibody showing positivity in neoplastic cells (×40).

**Figure 5:**
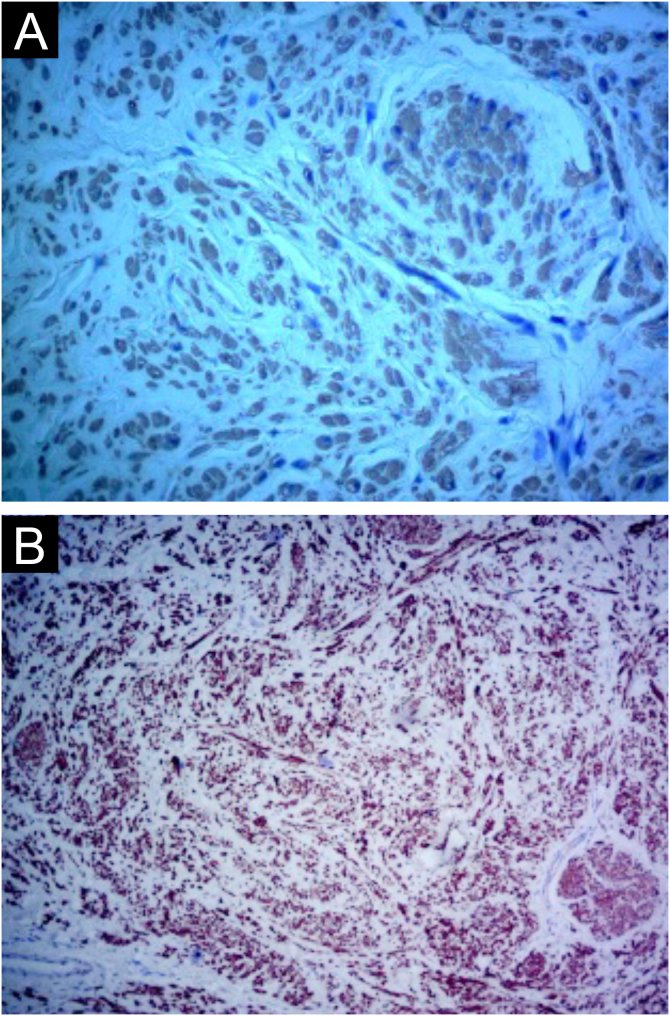
Case 2. (A) Immunohistochemistry with smooth muscle actin antibody, showing positivity in neoplastic cells (×400). (B) Immunohistochemistry with desmin antibody, showing positivity in neoplastic cells (×400).

Histologically, the lesions presented as a proliferation of smooth muscle cells forming intersecting bundles with sparse intervening connective tissue. The cells had elongated nuclei and tapered eosinophilic cytoplasm (Figure 2, Figure 3). No cytological atypia or mitotic figures were observed. Immunohistochemistry was positive for desmin and smooth muscle actin in both cases (Figure 4, Figure 5). It is important to differentiate this from cutaneous leiomyosarcoma, an entity that demonstrates hypercellularity, cellular atypia, and a high mitotic index. The diffuse expression of smooth muscle markers such as desmin and SMA also helps identify leiomyomas and distinguish them from other entities.

Cutaneous leiomyomas are rare benign smooth muscle tumors, with few cases reported since their first description by Virchow in 1854.^1^, ^2^ They have a heterogeneous clinical presentation and are commonly overlooked as a diagnostic hypothesis.^3^ Cutaneous leiomyomas can be classified into three categories according to the muscle fibers of origin: pilar leiomyomas (originating from the muscle fibers of the arrector pili muscle and the most common subtype), angioleiomyoma (originating from the tunica media of blood vessels, the second most prevalent), and dartoic or genital leiomyoma (originating from the smooth muscles of the scrotum, nipple, areola, or vulva and the rarest presentation). Among genital leiomyomas, those located on the nipples and areolas are even rarer, with little literature available on them.^4^, ^5^

Leiomyoma of the nipple is a very rare benign neoplasm, first described by Virchow in 1854.^2^ According to Kaufman et al., the leiomyoma originates in the nipple and parenchyma from the smooth muscle cells surrounding the capillaries in the subcutaneous tissue of the breast.^6^ Diaz-Arias et al. suggested that the origin of these tumors may be (a) teratoid, originating from extensive overgrowth of myomatous elements, (b) embryonic displacement of the smooth muscle of the nipple, (c) angiomatous smooth muscle, (d) multipotent mesenchymal cells, and (e) myoepithelial cells.^7^ Its presentation is heterogeneous, depending on its histological type, and can open up a range of possible differential diagnoses, especially if it is a solitary lesion. Genital leiomyoma most characteristically presents as a single, painless, sporadically pedunculated, papular-nodular lesion, smaller than two centimeters, mainly in the scrotum, penis, and vulva. It can affect the nipple-areolar complex and rarely involves both breasts.^3^, ^8^

Clinically, cutaneous leiomyomas, especially pilar leiomyomas, generally present as firm papules or nodules, which may be multiple or single, flesh-colored or pink, and range in size from 0.2 mm to 2 cm. They may present with increased sensitivity and even pain due to muscle contraction and compression of local nerve fibers. If multiple, they may be organized along Blaschko lines^8^, ^9^ and should raise clinical suspicion of Hereditary Leiomyomatosis and Renal Cell Carcinoma (HLRCC) syndrome, a rare autosomal dominant hereditary disease caused by a heterozygous germline mutation in the gene encoding Fumarate Hydratase (FH) and characterized by the risk of developing cutaneous pilar leiomyomas, uterine leiomyomas, and papillary renal cell carcinoma type 2.^10^

Surgical removal is considered a curative approach for cutaneous leiomyomas. Other forms of treatment include medication options aimed at symptomatic control, such as calcium channel blockers, or destructive methods such as cryotherapy or laser therapy.^9^

Cutaneous leiomyomas are considered rare benign smooth muscle tumors and are classified according to the muscle fibers of origin, with genital leiomyomas being the least common type. This report describes two cases in which their location in the mammary areola makes them even rarer.

## ORCID IDs

Neusa Yuriko Sakai Valente: 0000-0002-8065-2695

Thaiz Brandão Cosac: 0009-0002-7440-0077

## Financial support

None declared.

## Authors' contributions

Mariana Abdo de Almeida: Approval of the final version of the manuscript; design and planning of the study; drafting and editing of the manuscript; collection, analysis, and interpretation of data; effective participation in research orientation; intellectual participation in the propaedeutic and/or therapeutic conduct of the studied cases; critical review of the literature; critical review of the manuscript.

Neusa Yuriko Sakai Valente: Approval of the final version of the manuscript; design and planning of the study; drafting and editing of the manuscript; collection, analysis, and interpretation of data; effective participation in research orientation; intellectual participation in the propaedeutic and/or therapeutic conduct of the studied cases; critical review of the literature; critical review of the manuscript.

Thaiz Brandão Cosac: Approval of the final version of the manuscript; design and planning of the study; drafting and editing of the manuscript; collection, analysis, and interpretation of data; intellectual participation in the propaedeutic and/or therapeutic conduct of the studied cases; critical review of the literature; critical review of the manuscript.

## Research data availability

Not applicable.

## Conflicts of interest

None declared.
